# The leader intron of *AtMHX *can elicit, in the absence of splicing, low-level intron-mediated enhancement that depends on the internal intron sequence

**DOI:** 10.1186/1471-2229-10-93

**Published:** 2010-05-20

**Authors:** Tsofit Akua, Irina Berezin, Orit Shaul

**Affiliations:** 1 The Mina and Everard Goodman Faculty of Life Sciences, Bar-Ilan University, Ramat-Gan, Israel

## Abstract

**Background:**

Introns stimulate gene expression in a wide range of organisms by increasing the levels of mature mRNA, without affecting mRNA stability. Although introns sometimes function as transcriptional enhancers, they usually stimulate expression by a process termed intron-mediated enhancement (IME). The mechanism of IME is largely unknown. While splicing *per se *is not sufficient for IME, as evident from the fact that not all introns increase expression, it is not clear yet whether splicing of the enhancing introns is *essential *for enhancement. The leader intron (LI) of the *Arabidopsis AtMHX *gene was previously shown to substantially increase the expression of the *AtMHX *promoter. Here we investigated whether this LI acts as a transcriptional enhancer and whether its splicing is essential for IME.

**Results:**

Expression in transformed *Arabidopsis *plants of an *AtMHX*::GUS construct from which the LI was eliminated was similar to a construct that included only the minimal promoter fused to GUS. Yet, almost no expression was seen in constructs that included the LI in addition to the minimal promoter or the LI inserted in various locations in the promoter. While the LI enhanced 272-fold the expression of the weak *AtMHX *promoter, only a 3-fold enhancement was observed for the strong CaMV 35S promoter. In the context of the *AtMHX *promoter, an unspliceable version of the LI that had mutated 5' and 3' splice sites mediated a low-level (5-fold) enhancement. Eliminating the internal 320 nt of the 416 nt unspliceable intron resulted in loss of ability to mediate low-level enhancement.

**Conclusions:**

Although *AtMHX *promoter shows almost no expression in the absence of its LI, this intron does not act as a transcriptional enhancer and is unable to support expression in the absence of the enhancer elements of the promoter. It is also shown that the same intron can have very different contributions to expression of different promoters. Our results also demonstrate that while splicing is essential for substantial IME, in the absence of splicing low-level enhancement can be obtained. Notably, it is shown that the internal intron sequence plays a significant role in mediating the low-level enhancement of unspliced introns.

## Background

Introns have been shown to stimulate gene expression in a wide range of organisms, including mammals, nematodes, insects, fungi, and plants (reviewed in [1]). Introns enhance gene expression by increasing the steady-state amount of mature mRNA in the cell [2], apparently without significantly changing mRNA stability [3,4]. There are two general ways by which introns can elevate mRNA levels: first, by acting as transcriptional enhancers or alternative promoters located within the introns, and second, by a process termed intron-mediated enhancement (IME) [1].

The mechanism of IME is largely unknown, and several processes have been suggested as being involved (reviewed in [1]). It was suggested that exon junction complex (EJC) proteins associated with spliced introns can facilitate mRNA export to the cytoplasm [5] and also promote ribosome association with the mRNA, thus increasing translation efficiency [6]. As indicated by nuclear run-on transcription experiments, IME does not involve a change in the rate of transcription initiation [7-9]. Different introns have different abilities to boost expression. Splicing *per se *is not sufficient for enhancement, since some efficiently spliced introns have little or no effect while others stimulate expression to a great extent. Most enhancing introns are first introns, and it was found that promoter-proximal introns are enriched in dispersed, redundant short sequences that elevate gene expression [10]. It was hypothesized that the signals present in introns render the transcription machinery more processive, increasing the likelihood that full-length mRNAs will accumulate [10]. According to this model, in the absence of these signals, the polymerase may tend to dissociate and produce truncated, rapidly degraded transcripts [10].

While splicing *per se *is not sufficient for IME (as evident from the fact that not all introns increase expression), it is not clear yet whether splicing of the enhancing introns is *essential *for enhancement. Intron splicing was reported to be essential for enhancement in the maize *Sh1 *[11], *Adh1 *[12], and *Hsp82 *[13] genes. However, when splicing of the *Arabidopsis TRP1 *(formerly called *PAT1*) first intron was prevented, it was still able to enhance expression to about 50% of the enhancement mediated by the intact intron [9,14]. It was proposed that the difference between the observations with the maize *Sh1 *and the *Arabidopsis TRP1 *introns might indicate a potential difference between the mechanism of IME in either these introns or these plants [1].

The 5' untranslated region (5'UTR) of AtMHX, an *Arabidopsis *vacuolar metal/proton exchanger [15-17], includes the first intron of the *AtMHX *gene. This 416 nt leader intron (LI) was shown to enhance expression 86-fold in transgenic *Arabidopsis *(ecotype C24) plants when compared to a reporter construct that included *AtMHX *promoter without this intron [18]. It was pointed out that because different introns can affect expression by different mechanisms, a complete understanding of IME will require a detailed characterization of the phenomenon using multiple introns, genes, and species [14]. Here we investigated whether the LI of *AtMHX *acts as a transcriptional enhancer and whether its splicing is essential for IME. We found that although almost no expression was seen in the absence of this intron, it did not act as a transcriptional enhancer and was unable to support expression in the absence of the enhancer elements of the promoter. While splicing was essential for substantial IME, the LI was able to elicit low-level enhancement of expression in the absence of splicing. The internal LI sequence played a crucial role in the low-level enhancement mediated by the unspliced intron.

## Results

### The LI does not act as a transcriptional enhancer or internal promoter

**Figure 1:**
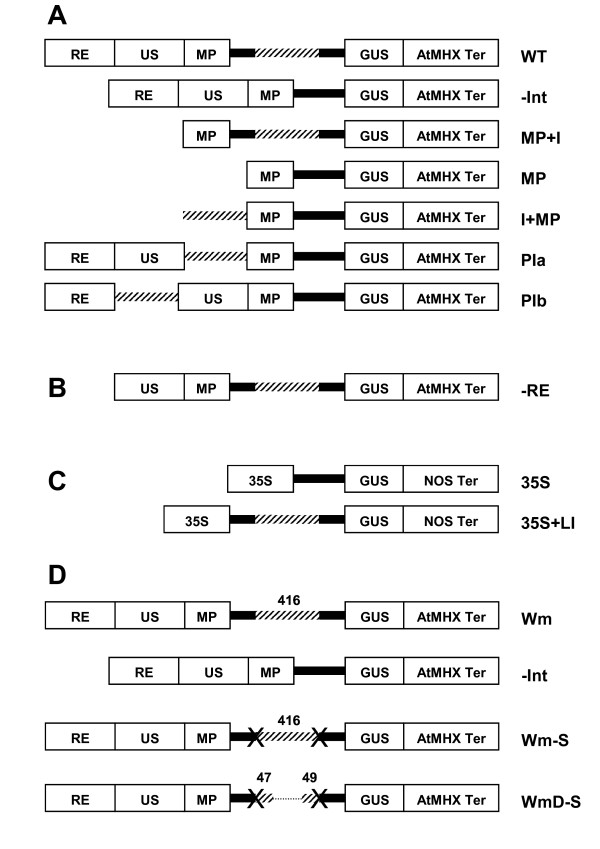
**Schematic representation of the constructs utilized**. The *AtMHX *promoter elements utilized were the repetitive element (RE), unique sequence (US), and minimal promoter (MP) (see Results and Methods for details about these elements). The solid line represents the 5'UTR of *AtMHX*, the diagonal lines represent the leader intron (LI) of *AtMHX*, and the dashed line represents the region eliminated from the LI. GUS - the coding sequence of β-glucuronidase; AtMHX ter - the terminator of the *AtMHX *gene; 35S - the CaMV 35S promoter; NOS ter - the terminator of the *Agrobacterium tumefaciens *nopaline synthase gene; X - an abolished splice site. The objective of each construct set is explained in the text.

In a few cases, plant introns were shown to act as transcriptional enhancers or internal promoters (reviewed in [1]). To distinguish whether an intron enhances expression by acting as a transcriptional enhancer or by IME, it is necessary to determine whether the intron can stimulate the expression of a gene that has only a minimal promoter, and whether the intron can enhance expression from outside the transcribed sequence [1]. To address this subject, the series of constructs shown in Figure 1A was created. The basic construct (WT) included GUS under the control of the regulatory regions of *AtMHX *- the promoter, 5'UTR, LI, and terminator. The -Int (minus intron) construct lacked the LI. The promoter is composed of three regions (Figure 1A): (i) a repetitive element (RE) of 530 bp [18]; (ii) a unique sequence (US) of 494 bp; and (iii) a minimal promoter of 78 bp, which includes the TATA and CAAT boxes [17]. In construct MP+I, all the promoter regions were removed except the minimal promoter. Thus, if the LI acts as a downstream transcriptional enhancer or includes an internal promoter, this construct should be expressed. Construct MP is a control that includes only the minimal promoter without the LI. In the other constructs, the LI was moved outside the transcribed sequence. In construct I+MP the LI is upstream to the minimal promoter, in construct PIa (promoter-intron a) the other promoter regions are also present upstream to the LI, and in construct PIb the LI is localized between the RE and US promoter regions.

**Figure 2:**
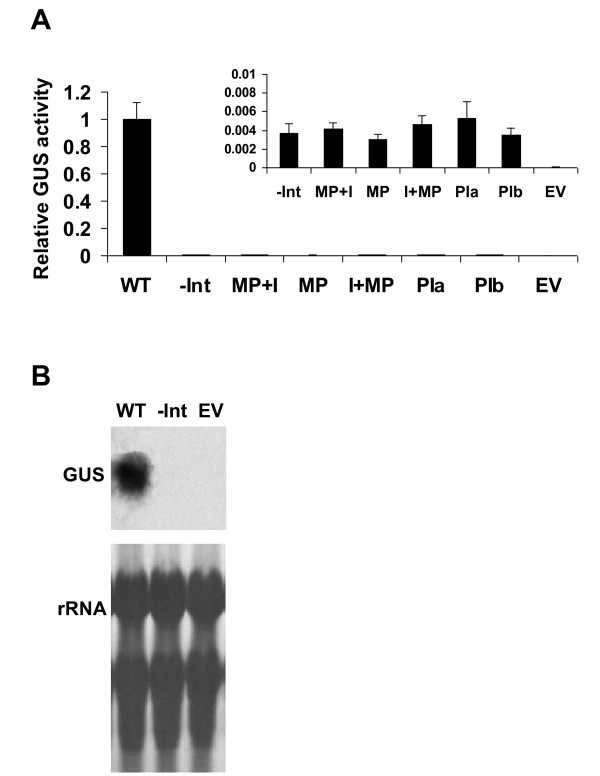
**The LI does not act as a transcriptional enhancer**. A. Mean and standard errors of relative GUS activity in mixtures including an equal number of T2 seedlings from 13 independent transformants of each construct. GUS activity of the WT construct was assigned the value of 1. EV, plants expressing the empty vector. The internal graph shows relative GUS activity compared to the WT construct on a smaller scale in plants expressing the constructs that mediated low GUS activity. Statistical analysis (*t*-test) revealed a highly significant difference (*p *< 0.01) between plants expressing the WT and all other constructs, but no significant difference was revealed between plants expressing the -Int and the MP+I, MP, I+MP, PIa, or PIb constructs (*p *> 0.05). B. Representative Northern blot results with the GUS probe. rRNA, ribosomal RNA.

All the constructs shown in Figure 1A were stably transformed into *Arabidopsis thaliana *ecotype Col-0 plants. GUS activity was determined in a mixture including an equal number of T2 seedlings from 13 independent transformants of each construct. We previously observed that there is no significant correlation between the number of transgene copies in independent transformants of the same construct and the level of expression [19]. Thus, for each of the constructs created in this study, 13 to 20 independent transformants were utilized for expression analysis. Compared to the WT construct, all the other constructs showed much lower GUS expression (Figure 2A). GUS activity in plants with lower expression levels is shown on a smaller scale in the internal graph. The extent of enhancement gained by the LI, that is, the ratio between the expression of the WT and -Int constructs, was 272-fold in the transformed *Arabidopsis *(ecotype Col-0) plants (a value of 86-fold was previously observed in transformed *Arabidopsis *ecotype C24 plants [18]). This calculation was valid since GUS activity in the -Int construct, although very low, was still higher than that of plants expressing the empty vector (EV) (Figure 2A).

The expression level seen after eliminating the LI (the -Int construct) was similar to that of construct MP+I, in which all promoter elements except the minimal promoter were removed (Figure 2A). Thus, the 1102 bp upstream to the transcriptional start site of *AtMHX *could not elicit expression higher than that of the minimal promoter in the absence of the LI. Still, the LI was unable to support expression by itself in the absence of the enhancer elements of the promoter, as demonstrated by the low expression of construct MP+I. Constructs I+MP, PIa, and PIb demonstrated that the LI was not able to support expression when localized outside the transcribed region, even if the other promoter elements were present. The -RE construct shown in Figure 1B could serve as a control to construct PIb. Construct -RE was not examined here but was previously shown to be expressed to about half the level of the full promoter [18]. This indicated that the regulatory regions downstream the LI in construct PIb (US and MP) were sufficient as enhancer elements when the LI was present in its native position. Altogether, these data showed that the LI of *AtMHX *does not act as a transcriptional enhancer or internal promoter, and that its presence in the transcribed region is essential for expression. Thus, this LI acts by IME. Although almost no expression was seen in the absence of this LI, it was unable to support expression when the enhancer elements of the promoter were deficient.

The levels of GUS transcript in plants expressing the -Int construct were below the detection limit in Northern blot analysis of the transformed plants (Figure 2B). This confirmed that the enhancement resulted from an increase in the transcript levels, as typical for IME (see Introduction).

### The LI of *AtMHX *enhances 3-fold the expression of the CaMV 35S promoter

**Figure 3:**
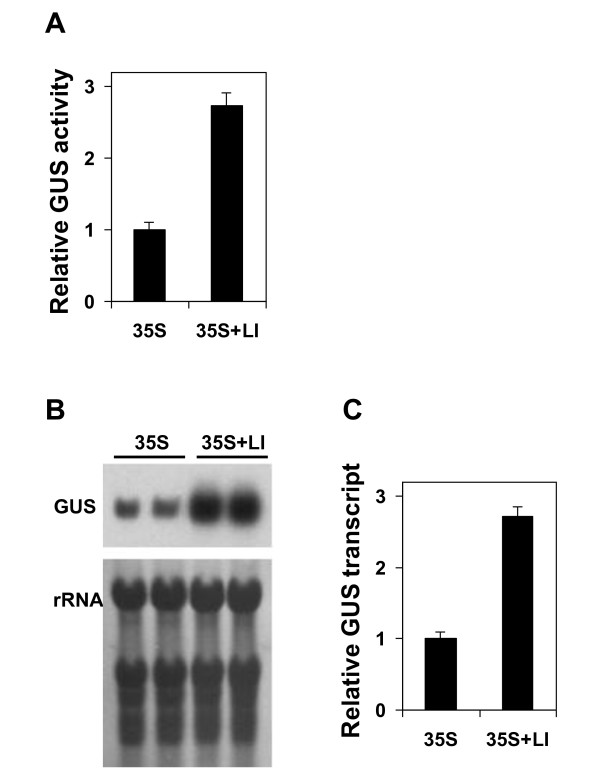
**A 3-fold increase in expression level is seen in the context of the 35S promoter**. A. Mean and standard errors of relative GUS activity in six biological replicates, each including an equal number of T2 seedlings from 20 independent transformants of each construct. GUS activity of the 35S construct was assigned the value of 1. B. Representative Northern blot results with the GUS probe. rRNA, ribosomal RNA. C. Mean and standard errors of relative GUS transcript levels in the six biological replicates. Quantification of band densities on gel was performed with the ImageJ program. The data are presented relative to the transcript level of the 35S construct.

Since *AtMHX *promoter shows almost no expression in the absence of its LI, it was interesting to learn how this intron affects the expression of a promoter that is well expressed on its own. To address this, it was investigated if the LI of *AtMHX *can enhance the expression of the strong CaMV 35S promoter. The constructs used included GUS under the control of the CaMV 35S promoter and NOS terminator, as well as the 5'UTR of *AtMHX *that either included (35S+LI) or did not include (35S) the LI (Figure 1C). The two constructs were stably transformed to *Arabidopsis *(Col-0) plants, and 20 independent transformants were collected and analyzed for each construct. Figure 3 shows the results of a typical experiment. Inclusion of the LI resulted in a 2.73-fold increase in GUS activity (Figure 3A). A 2.72-fold increase was observed in GUS transcript levels (Figure 3B, C), indicating that the enhancement resulted from an increase in the steady-state transcript levels.

Thus, the LI of *AtMHX *was able to enhance the expression mediated by the strong CaMV 35S promoter by about 3-fold. This was much lower than the extent of IME gained with the *AtMHX *promoter, which was about 270-fold. The absolute values of GUS activity in plants expressing constructs 35S (without the LI) and WT (*AtMHX *promoter including the LI) were 320 and 2 milli units ^. ^mg protein^-1^, respectively. That is, even with its LI, the *AtMHX *promoter is much weaker than the CaMV 35S promoter (however, the *AtMHX *promoter is not constitutive and shows increased expression at specific sites). Additional experiments were carried out in the context of the *AtMHX *promoter in order to learn more about the requirements for IME in the natural combination of promoter and intron, in which almost no expression is seen in the absence of the intron.

### Splicing was essential for substantial enhancement whereas an unspliced intron mediated low-level enhancement that was dependent on the internal intron sequence

There were different conclusions with respect to the question if splicing is essential for IME (see Introduction). To investigate if splicing is essential for the enhancement mediated by the LI of *AtMHX*, the Wm-S (WT modified minus splicing) construct was created (Figure 1D). This construct included mutated 5' and 3' splice sites, as well as several other essential modifications (see details below). The Wm (WT modified) was a control construct whose sequence was identical to Wm-S except the modifications at the vicinity of the splice sites. It was also desired to determine whether the internal region of the unspliceable intron plays a role in a potential enhancement. For this, the internal 320 nt of the Wm-S construct were deleted to create the WmD-S construct (Figure 1D). In the deleted construct, only the proximal 47 and 49 nt were left from the 5' and 3' borders, respectively, of the Wm-S intron, thus creating a 96 nt intron (the native LI is 416 nt) having mutated splice sites.

**Figure 4:**
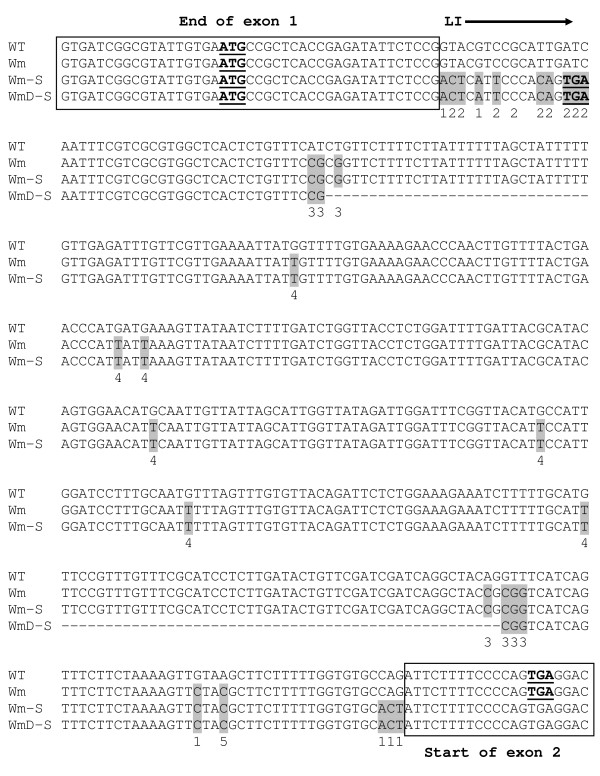
**The modifications introduced in the different LIs**. Boxed, partial sequence of the 5'UTR exons that border the LIs. Exon sequence was not altered in any of the constructs. The modifications introduced into the Wm and Wm-S constructs are highlighted in gray. The underlying numbers indicate the purpose of each modification: 1 - eliminating the main and potential cryptic splice sites, 2 - maintaining the length, deduced amino-acid sequence and predicted secondary structure of the natural uORF found in the 5'UTR of *AtMHX *[the first (ATG) and last (TGA) codons of the uORF of each construct are bold and underlined], 3 - creating SacII sites for obtaining the WmD-S from the Wm-S construct, 4 - eliminating the internal ATG codons, and 5 - eliminating a HindIII site that interfered with cloning into the binary vector.

As mentioned, it was necessary to introduce several other modifications into the Wm-S and WmD-S constructs (Figure 4). Two point mutations were introduced to prevent cryptic splicing of the retained introns. It was also necessary to introduce seven point mutations that eliminated all internal ATG codons. Since the unspliced LI will remain as part of the 5'UTR, the presence of ATG codons in it may inhibit the translation of the main ORF [20] and/or result in the formation of premature termination codons (PTCs). Such PTCs may lead to transcript degradation by the nonsense-mediated mRNA decay (NMD) quality-control mechanism, which degrades transcripts bearing PTCs [21]. Few other modifications were designed to maintain in the unspliced transcript the natural upstream open-reading frame (uORF) included in the 5'UTR of *AtMHX*. Similar to the native *AtMHX *transcript, in the WT, Wm and -Int constructs this uORF initiated in the first exon of the 5'UTR and terminated in the second exon (Figure 4). In constructs with eliminated splice sites (Wm-S and WmD-S), the uORF terminated within the unspliced intron, which was mutated to encode an uORF identical to that of the other constructs. Thus, all the constructs used in this study included an identical version of the uORF. Few other mutations were designed to eliminate a HindIII site and to introduce two SacII sites for the creation of the WmD-S construct.

**Figure 5:**
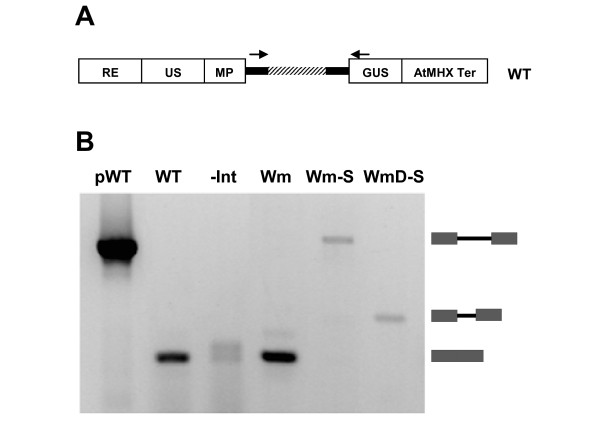
**Determination of splicing efficiency**. A. The primers used for RT-PCR are indicated on the illustration of the WT construct. The forward and reverse primers were derived from the first exon of the 5'UTR and from the GUS coding sequence, respectively. B. The results of the RT-PCR analysis. The analysis was not quantitative but qualitative (that is, the analysis shows the size of each transcript, but band intensity do not accurately reflect the relative abundance of the different transcripts). All the templates were cDNA except pWT, which was the plasmid that included the WT construct. The product of pWT thus indicated the size of unspliced WT transcript. The boxes separated by a long or short line indicate the expected size of unspliced transcripts including either the full or deleted LI, respectively. The two adjacent boxes indicate the size of correctly spliced transcripts.

All these constructs were stably transformed to *Arabidopsis *Col-0 plants. To verify that the WT and Wm constructs were spliced correctly while the Wm-S and WmD-S remained unspliced, the transformed plants were subjected to reverse transcription polymerase chain reaction (RT-PCR) analysis with the primers indicated in Figure 5A. Plants expressing the WT and Wm constructs showed a product with the expected spliced size (Figure 5B), which was smaller than the unspliced PCR product obtained from the plasmid including the WT construct (pWT). Inevitably, plants expressing the -Int construct showed a product with the expected unspliced size (and a somewhat larger non-specific product). The two -S constructs, in which splice sites were eliminated, showed a product with their expected unspliced size (which was smaller for WmD-S than for Wm-S).

**Figure 6:**
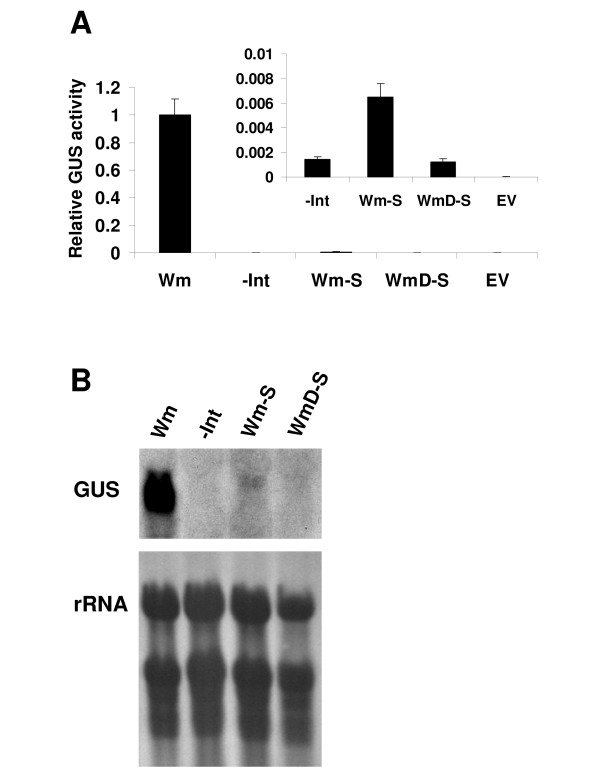
**The unspliced LI mediates low-level enhancement**. A. Mean and standard errors of relative GUS activity in mixtures including an equal number of T2 seedlings from 13 independent transformants of each construct. GUS activity of the Wm construct was assigned the value of 1. EV, plants expressing the empty vector. The internal graph shows relative GUS activity compared to the Wm construct on a smaller scale in plants expressing the constructs that mediated low GUS activity. Statistical analysis (*t*-test) revealed a highly significant difference (*p *< 0.01) between plants expressing the Wm and all other constructs. A highly significant difference (*p *< 0.01) was also observed between plants expressing the -Int and the Wm-S constructs, but not between plants expressing the -Int and the WmD-S construct. B. Representative Northern blot results with the GUS probe. rRNA, ribosomal RNA.

GUS activity was determined in a mixture including an equal number of T2 seedlings from at least 13 independent transformants of each construct (Figure 6A). GUS activity in plants with lower expression level is shown on a smaller scale in the internal graph. Eliminating the splicing ability of the LI in the Wm-S construct resulted in a significant reduction in expression as compared to the Wm construct. Still, the unspliceable intron of the Wm-S construct mediated low-level enhancement of GUS expression. This was evident from the fact that GUS activity of plants expressing the Wm-S construct was about 5-fold higher compared to the -Int construct (internal graph in Figure 6A). Statistical analysis (*t*-test) showed that the difference in expression between the -Int and Wm-S constructs was highly significant (*p *< 0.01). Figure 6B shows the results of Northern blot hybridization with the GUS probe of RNA extracted from these plants. While GUS transcript levels were below the detection limit in plants expressing the -Int construct, a weak band was observed in the Wm-S construct. The size of this band was somewhat higher than that of the Wm construct since this transcript included the additional 416 nt of the LI. Thus, the retained intron was able to mediate low-level enhancement. These results indicated that while intron splicing was essential for substantial IME, low-level enhancement could be achieved in the absence of splicing.

Interestingly, the loss of a significant part of the internal LI sequence in the WmD-S construct resulted in a loss of ability to mediate low-level enhancement. The WmD-S construct included the proximal 47 and 49 5' and 3' nt, respectively, of the Wm-S construct, but lacked the internal 320 nt of the latter construct. The resulting 96 nt unspliceable intron mediated GUS activity that was similar to that of the -Int construct, and its GUS transcript was below the detection limit (Figure 6).

## Discussion

The mechanism of IME is not well understood. To identify the rules that govern this process, it is important to obtain information from multiple introns. The results shown here indicated that the LI of *AtMHX *does not act as a transcriptional enhancer or internal promoter, and that its presence in the transcribed region is essential for enhancement. Thus, this LI acts by IME. Although almost no expression was seen without this intron, it was unable to support expression in the absence of the enhancer elements of the promoter. Previous work showed that histochemical GUS staining was below the detection limit in plants expressing the -Int construct, whereas staining could be clearly seen in specific tissues of plants expressing GUS under the control of *AtMHX *promoter together with the LI ([18] and data not shown). Our current data suggest that the enhancers that determine the expression in these specific tissues are located in the promoter, while the LI merely enhances expression above the detection limit.

Even with its LI, the *AtMHX *promoter is much weaker than the CaMV 35S promoter (still, it must be taken into consideration that in contrast to the CaMV 35S promoter, the *AtMHX *promoter is not constitutive and shows increased expression at specific sites [18]). While in its natural position in the context of the *AtMHX *promoter the LI enhanced expression approximately 270-fold, only a 3-fold enhancement was seen in the context of the CaMV 35S promoter. This demonstrates the stronger contribution of this enhancing intron to a weak promoter.

While splicing *per se *is not sufficient for enhancement, the question whether splicing of enhancing introns is *essential *for enhancement is not clear at present (see Introduction). The combination of *AtMHX *promoter and LI provides an interesting system in which the intron is virtually essential for expression. It was reported that splicing of the first introns of the maize *Hsp8 *and *Adh1 *genes was essential for enhancement [12,13]. However, in these studies, as noted by Rose and Beliakoff [9], the retained introns might have caused NMD due to internal ATG or termination codons. In two other reports, the ability of the retained introns to cause NMD was eliminated. In the first of these studies, ATG codons within the maize *Sh1 *intron, which was located in the 5'UTR, were eliminated by point mutations, and it was concluded that splicing of this intron was essential for enhancement [11]. In the second study, potential termination codons were eliminated from the retained *Arabidopsis TRP1 *intron, which was located in the coding sequence [9,14]. It was concluded that splicing of this intron was not essential for enhancement, since preventing splicing resulted in 50% of the enhancement mediated by the intact intron. It was suggested that the difference between the observations with the maize *Sh1 *and *Arabidopsis TRP1 *introns might indicate a potential difference in the mechanism of IME between either these introns or these plants [1].

However, considering the results presented for the maize *Sh1 *and *Arabidopsis TRP1 *introns together with the results reported here, a general picture emerges. In the current study, the ability of the retained intron to cause NMD was eliminated by point mutations that eliminated its internal ATG codons. The WT and Wm-S constructs mediated a 270- and 5-fold enhancement of expression, respectively, compared to the -Int construct. For the maize *Sh1 *intron, there was a 25-44-fold enhancement with spliced introns, and a 2-fold enhancement when the splice sites were mutated [11]. For the *Arabidopsis TRP1 *intron there was an approximately 5- and 2.5-fold enhancement with spliced and unspliced introns, respectively [9,14]. Altogether, these data indicate that in the absence of splicing, low-level enhancement can be achieved while splicing is essential for substantial enhancement. In the three studies discussed, low-level enhancement in the absence of splicing ranged from 2- to 5-fold. The biggest value (the 5-fold enhancement reported here for the Wm-S construct) was obtained in the combination of intron and promoter in which full enhancement was highest (270-fold for the WT over the -Int construct).

In the current study, achievement of low-level enhancement by the unspliced LI was dependent on the presence of a significant part of the internal intron sequence. Compared to the Wm-S construct, which mediated 5-fold enhancement, the WmD-S construct, in which 320 nt of the internal intron sequence were eliminated, lost its ability to mediate low-level enhancement. For comparison, an internal deletion of 32 nt in the 108 nt *Arabidopsis TRP1 *intron that had a mutated 5' splice site did not abolish the ability of this unspliceable intron to mediate low-level enhancement [14]. An internal deletion of 47 nt in the same *TRP1 *intron, which rendered it unspliceable due to its short size (the lower limit for intron splicing in plants is 70-73 nt [22]), also did not eliminate its ability to mediate low-level enhancement [9]. However, in the latter case the resulting intron had intact splice sites. The presence of intact splice sites as well as a potential branch point (even if the intron was rendered unspliceable due to other factors) was shown to be more important for low-level enhancement mediated by the *TRP1 *intron than the presence of a significant part of the internal intron sequence [9,14]. Based on these findings, it was suggested that the mechanism of IME requires that the splicing machinery will be at least partly assembled onto an intron, even if it is unable to complete its task [1]. Interestingly, our results showed that the internal sequence of the *AtMHX *LI played a crucial role in mediating the low-level enhancement of the mutated intron that had abolished splice sites.

What could be the reason for these findings? It seems that besides elements such as splice sites and a potential branch point, other internal elements of enhancing introns can stimulate low-level enhancement in the absence of splicing. Possible candidates for these internal elements are redundant, unspecified short sequences enriched in promoter-proximal introns, whose presence in spliced introns was shown to elevate gene expression [10]. It is possible that these elements contribute not only to the substantial enhancement mediated by spliced introns, but also to the low-level enhancement mediated by unspliced introns. According to this hypothesis, in the case of the enhancing-capable short (108 nt) *TRP1 *intron, omitting 32 nt [14] of its internal sequence possibly kept enough of the sequences required for enhancement to allow low-level enhancement in the presence of the mutated splice sites. When 47 nt of this intron were deleted [9], it is possible that, as previously suggested [1], the retained intact splice sites allowed partial assembly of the splicing machinery that enabled enhancement to a limited extent. In the current study, omitting 320 nt of the internal *AtMHX *LI sequence apparently eliminated internal sequences that were essential for low-level enhancement in the absence of intact splice sites. To evaluate our suggestion about the contribution of promoter-proximal intron sequences to low-level enhancement in the absence of splicing or intact splice sites, we utilized the IMEter algorithm [23]. This algorithm assigns an "IMEter score", which indicates to which extent the sequence of a given intron resembles that of promoter-proximal introns in *Arabidopsis *[10]. The IMEter scores of the introns in the Wm, Wm-S, and WmD-S constructs were 37.7, 34.7, and 6.9, respectively. Thus, the IME score, which was previously shown to correlate with the degree of substantial enhancement mediated by spliced introns [10], correlated here with the ability of unspliced introns that had abolished splice sites to cause low-level enhancement. Further experiments will be however necessary to determine whether the sequences required for low-level enhancement in the absence of splicing or intact splice sites actually match the redundant short sequences necessary for substantial enhancement activation by spliced introns.

## Conclusions

Although *AtMHX *promoter shows almost no expression in the absence of its LI, this intron does not act as a transcriptional enhancer and is unable to support expression in the absence of the enhancer elements of the promoter. It is also shown that the same intron can have different contributions to expression of different promoters. This work also demonstrates that while splicing is essential for substantial IME, in the absence of splicing low-level enhancement can be obtained. Notably, it is shown that the internal intron sequence plays a significant role in mediating the low-level enhancement of unspliceable introns.

## Methods

### Plant transformation and growth conditions

*Arabidopsis thaliana *(L.) (ecotype Col-0) plants were transformed using the floral dip technique [24] and grown in a greenhouse in a photoperiod of 16 h light and 8 h dark. For expression analysis, T2 seedlings were germinated on MS plates containing kanamycin, and grown for two weeks in a climate-controlled growth room.

### Construction of plasmids

Cloning of the regulatory and transcribed regions of *AtMHX *[TAIR:AT2G47600], including the promoter, terminator, 5'UTR, and LI was described [17,18]. Cloning GUS under control of *AtMHX *promoter, terminator, 5'UTR, and LI was also described (the construct called Full in [18] is called here WT). Construct -Int is similar to WT but includes the mature 5'UTR without the LI. Constructs MP+I and MP were created by amplification of constructs WT and -Int, respectively, with the following primers: forward 5'-GCGAGCATGCCCCCGTCGACGATACAATAATTGAAGTGTGTCAT-3' and reverse 5'-TTAGGCCATGGTAACTTATTCAAA-3'. The two products were digested with SpHI and NcoI and cloned into the corresponding sites of construct WT. To create constructs I+MP, PIa, and PIb, the LI sequence was amplified by PCR and cloned into the positions indicated in Figure 1A. The minimal promoter included 78 bp upstream to the transcription start site of *AtMHX *[17], the region called US (unique sequence) included the 494 bp upstream to the minimal promoter, and the region called RE (repetitive element) included the 530 bp upstream to the US. To create construct 35S, the NcoI-Bsp119I fragment (including part of the GUS-intron sequence) of plasmid pWT-GUS [25] was replaced with the corresponding restriction fragment of the GUS-coding sequence that did not include an intron from plasmid pJD330 (a kind gift of DR Gallie). To create construct 35S+LI, the mature 5'UTR present in construct 35S was replaced with the 5'UTR of *AtMHX *that included the LI, which was amplified by PCR from the WT construct. The intron of the Wm-S construct as well as short fragments of the two adjacent exons were synthesized by GenScript USA Inc. (Piscataway, NJ). The two adjacent exons included Mva1269I and HincII sites that enabled cloning of the modified LI into the corresponding sites of construct WT. Construct Wm was crated by amplifying the Wm-S intron with the following primers, which restored the native sequence at the vicinity of the splice sites: forward 5'-GAATGCCGCTCACCGAGATATTCTCCGGTACGTCCGCATTGATCAATTTCGTCGCGT-3' and reverse 5'-GTCAACTGAACACTTGTCCTCACTGGGGAAAAGAATCTGGCACACCAAA-3'. This was followed by digestion with Mva1269I and HincII and cloning into the corresponding sites of construct Wm-S. The WmD-S construct was created by digestion of the Wm-S construct with SacII and self ligation. The secondary structure of the resulting uORFs was predicted as described [26] and confirmed to be similar to that of the WT construct. All chimeric genes were verified by sequencing, cloned into the binary vector pGA492 [27], immobilized into *Agrobacterium *EHA105 [28], and used for plant transformation.

### RNA extraction and Northern blot analysis

Total RNA was extracted with TRI-Reagent (Sigma, St Louis, MO) according to the manufacturer's instructions. RNA samples were denatured with glyoxal (Sigma, St Louis, MO) and fractionated on 1% agarose gels as described [29]. Gel preparation and fractionation were carried out with 10 mM NaPi buffer, pH 7.0. The gels were blotted onto a Zeta-Probe GT membrane (Bio-Rad) with 25 mM NaPi buffer, pH 7.0. RNA was fixed by UV and membranes were stained in 0.02% methylene blue in 0.3 M sodium acetate (pH 5.5) and rinsed in H_2_O. Hybridization was carried out using the DIG-labeling system (Roche Diagnostics GmbH) according to the manufacturer's instructions.

### Preparation of cDNA and RT-PCR

RNA was treated with DNase I, and then the DNase I was removed using Ambion's AM1906 kit. A preliminary PCR reaction with the same primers that were subsequently used for RT-PCR was conducted to verify that no DNA remained. Preparation of cDNA was carried out using an oligo dT primer and M-MuLV reverse transcriptase (Cat. no. EP0441, Fermentas). Two microliters of the cDNA were utilized as templates for RT-PCR analysis with the following primers: forward primer 5'-GCAGGATCCACGCTTGACCGATTC-3' and reverse primer 5'- TTCGCGATCCAGACTG-3'. PCR conditions were as follow: an initial cycle at 94°C for 3 min, followed by 30 cycles at 94°C for 30 sec, at 50°C for 30 sec, at 72°C for 45 sec, and a final cycle at 72°C for 10 min.

### Quantitative GUS analysis

For quantitative measurement of GUS activity, plant material was ground in liquid nitrogen and extracted in a buffer containing 50 mM NaPO_4_, pH 7.2, 1 mM Na_2_EDTA, 10 mM β-mercaptoethanol, and 10% (v/v) Triton X-100. Following centrifugation (5 min, 14,000 g, 4°C), samples of the supernatant were suspended in 250 μl extraction buffer including 1 mM (final concentration) of the fluorescent GUS substrate 4-methylumbelliferyl-β-D-glucuronide (MUG) (Duchefa Biochemie BV). GUS activity was assayed on a 96-well fluorescent plate-reader (Fluoroscan II, Lab Systems) with the excitation wavelength set at 350 nm and the emission wavelength at 460 nm. GUS activity (milli units ^. ^mg protein^-1^) was calculated from the slope of the line generated from measures taken at three-minute intervals during two hours, with respect to the slope of commercial pure GUS enzyme (Roche Diagnostics GmbH).

## Authors' contributions

TA carried out the molecular biology work, plant transformation and expression analysis. IB helped in the expression analysis. OS coordinated the project and prepared the manuscript. All authors read and approved the final manuscript.
